# 3D printing the pterygopalatine fossa: a negative space model of a complex structure

**DOI:** 10.1007/s00276-017-1916-x

**Published:** 2017-08-30

**Authors:** Ross Bannon, Shivani Parihar, Yiannis Skarparis, Ourania Varsou, Enis Cezayirli

**Affiliations:** School of Medicine, University of St Andrews, North Haugh, St Andrews, KY16 9TF Scotland

**Keywords:** Pterygopalatine fossa, 3D printing, Anatomy education, Model and simulation, 3D imaging techniques, Computed tomography

## Abstract

**Purpose:**

The pterygopalatine fossa is one of the most complex anatomical regions to understand. It is poorly visualized in cadaveric dissection and most textbooks rely on schematic depictions. We describe our approach to creating a low-cost, 3D model of the pterygopalatine fossa, including its associated canals and foramina, using an affordable “desktop” 3D printer.

**Methods:**

We used open source software to create a volume render of the pterygopalatine fossa from axial slices of a head computerised tomography scan. These data were then exported to a 3D printer to produce an anatomically accurate model.

**Results:**

The resulting ‘negative space’ model of the pterygopalatine fossa provides a useful and innovative aid for understanding the complex anatomical relationships of the pterygopalatine fossa.

**Conclusion:**

This model was designed primarily for medical students; however, it will also be of interest to postgraduates in ENT, ophthalmology, neurosurgery, and radiology. The technical process described may be replicated by other departments wishing to develop their own anatomical models whilst incurring minimal costs.

## Introduction

The pterygopalatine fossa (PPF) is a bony space located in the skull base that contains several important neurovascular structures. It is described as an “inverse pyramid” shaped space, located immediately posterior to the orbit. Its boundaries consist of the pterygomaxillary fissure laterally, the pterygoid plates of the sphenoid bone posteriorly, the perpendicular plate of the palatine bone medially, the sloping posterior surface of the maxillary bone anteriorly, and the inferior orbital fissure superiorly [17]. It contains the pterygopalatine ganglion; maxillary division of the trigeminal nerve, plus its branches; the maxillary artery (3rd part) plus its branches; emissary veins and a fatty matrix [5, 6]. Understanding this three-dimensional (3D) space is challenging: cadaveric dissection and two-dimensional (2D) textbook schematics do not allow full appreciation of its structure and communicating channels [16]. The PPF has been studied extensively using cross-sectional imaging in the form of computerised tomography (CT) and magnetic resonance imaging (MRI) [5, 6, 14, 17]. Despite these efforts, it remains an enigmatic structure for the student–anatomist and even aspiring surgeons and radiologists.

Three-dimensional (3D) printing, also known as rapid prototyping or additive manufacturing, encompasses several techniques devised to create 3D objects using computer data [3]. One such technique is fused deposition modelling (FDM). This involves heating a thermoplastic material to a semi-molten state, prior to using an extruder device to deposit consecutive layers of the material onto the bed of the printer. The material solidifies as it cools and fan-assisted cooling is often used to facilitate this process [7]. FDM printers can process a variety of materials. Most commonly used are plastic polymers such as polylactic acid (PLA) and acrylonitrile butadiene styrene (ABS)—the same material used to produce LEGO^®^. However, these polymers can be combined with metals, ceramics, or even natural fibres such as wood to create hybrid materials. The printing of flexible plastics is now also a possibility [13]. This technology has become widely available in recent years, particularly with the advent of ‘desktop’ printers which can be purchased by household consumers.

The role of 3D printing in medicine is already well established. Digital Imaging and Communications in Medicine (DICOM) is the industry standard for medical imaging data sets, developed to ensure compatibility across applications and devices, such as picture archiving and communication system (PACS) clients [8]. By processing DICOM data from cross-sectional imaging studies using specialized software, 3D renders of anatomical or pathological structures can be produced. This process facilitates patient-specific treatments, from the production of moulds for dental implants to 3D printed fractured bones used for operative planning [2, 12]. The field of medical education has recently harnessed the power of 3D printing through the production of anatomically accurate replicas of prosections based on imaging data [11, 18]. In this study, McMenamin et al. also described printing negative spaces to demonstrate complex anatomy—this is illustrated through a 3D print of the air sinuses in a warthog skull. Further work by this department has included detailed prints of prosections of the orbit [1]. These studies used a powder infiltration printer that can produce multi-coloured models. This field is developing rapidly and is expected to revolutionise the way that we teach anatomy, especially if 3D printing is combined with the currently available anatomical atlases to produce models for educational purposes.

The aim of this study was to produce a low-cost 3D model of the pterygopalatine fossa using data extracted from a CT scan, open source (free) software, and a 3D printer. This report describes the process of model production, thus facilitating replication of our technique.

## Materials and methods

**Fig. 1:**
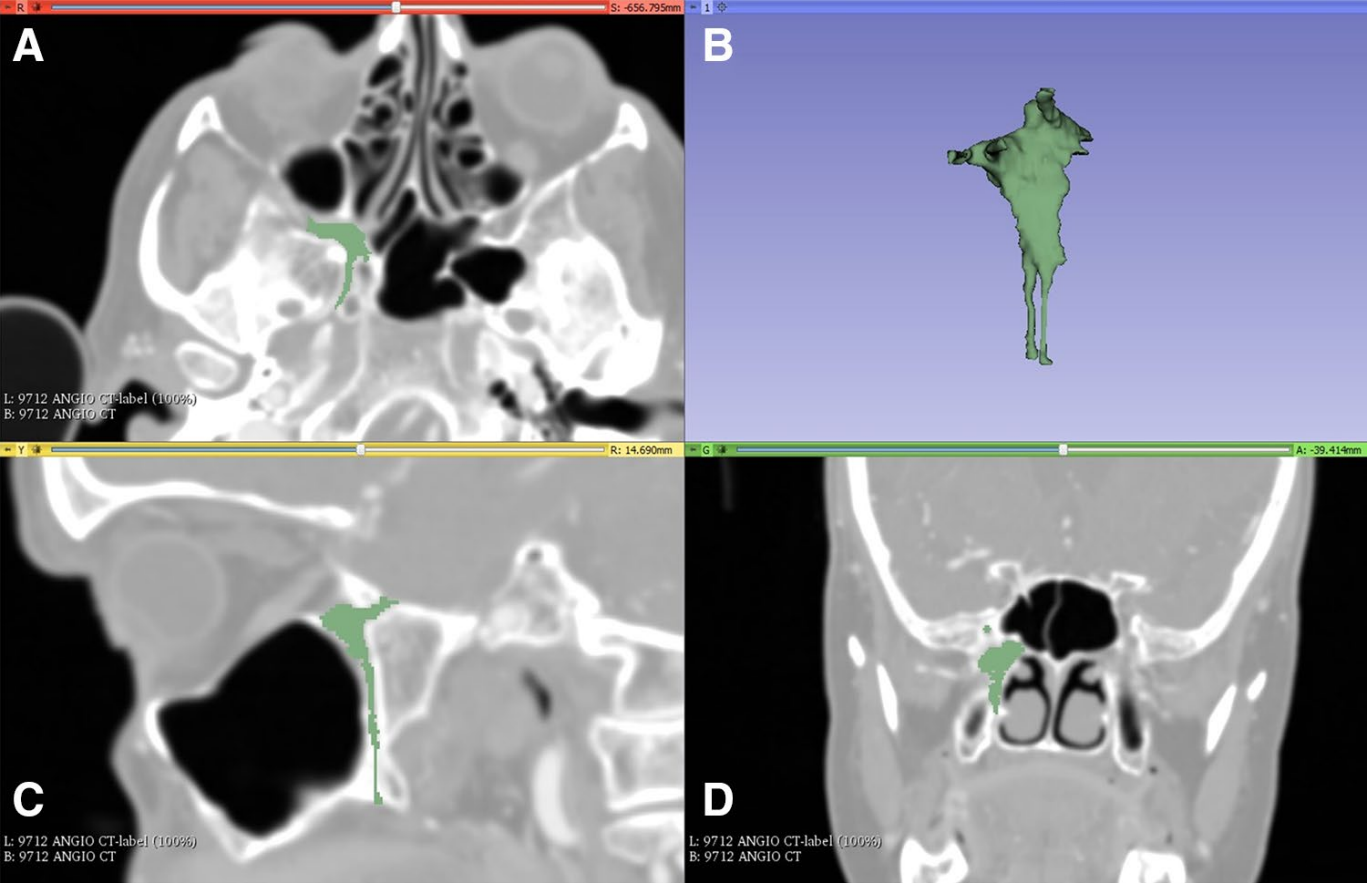
Process of manual segmentation in *3D Slicer*. **a** Axial slice showing the right PPF (including the pterygoid canal) which was highlighted using the draw tool. **b** 3D render synthesized from all highlighted slices. **c** Sagittal slice of the highlighted area showing the foramen rotundum and lesser palatine canal. **d** Coronal slice showing the bulk of the PPF and foramen rotundum

A sample computed tomography (CT) scan of the head DICOM data set was downloaded from the *Osirix* image database (http://www.osirix-viewer.com, Pixmeo SARL, Geneva)—a collection of anonymised data sets freely available for research and teaching purposes. This was loaded into *3D*-*Slicer* (http://www.slicer.org), an open source software platform used to handle DICOM images, for processing. Upon loading, a multiplanar reconstruction (MPR) of the data set allows the user to scroll through images in three axes: sagittal, coronal, and axial. Two approaches to creating a 3D render of a structure have been described. The first, volume rendering, uses the software program to select all data points of an equal intensity. The second, surface rendering or manual segmentation, requires an operator to highlight the desired structure in several slices [4]. Manual segmentation was completed by a single operator. This was accomplished using the draw tool in the program’s Editor Module to highlight all of the pixels that make up the space of the pterygopalatine fossa. This was performed in each axial slice, before using the program to create a 3D volume render of the space. Thus, a ‘negative space’ representation of the PPF is produced (Fig. 1). With reference to the previous imaging studies using CT [17] and ultrahigh-resolution MRI [14], we were able to highlight the foramen rotundum, pterygoid canal, palato-vaginal canal, and the greater and lesser palatine canals, enabling their inclusion in the render. The volume render was then exported as an object file (.obj).

**Fig. 2:**
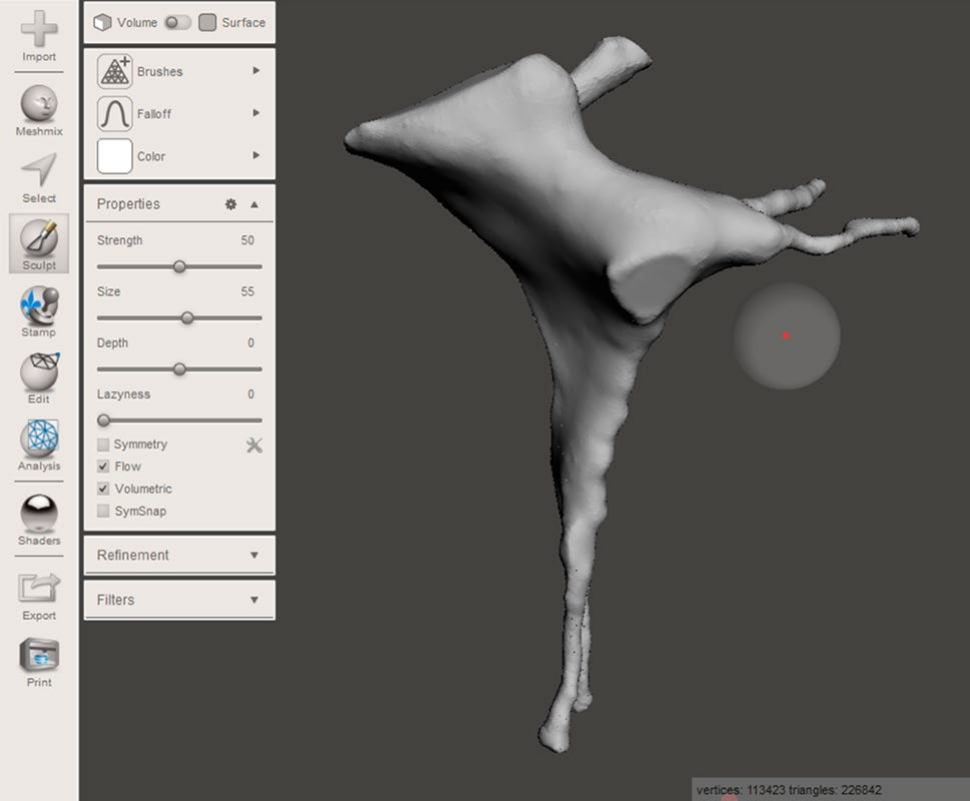
Process of model smoothing in *Meshmixer*. This step significantly increases the number of vertices that make up the model, increasing the file size

**Fig. 3:**
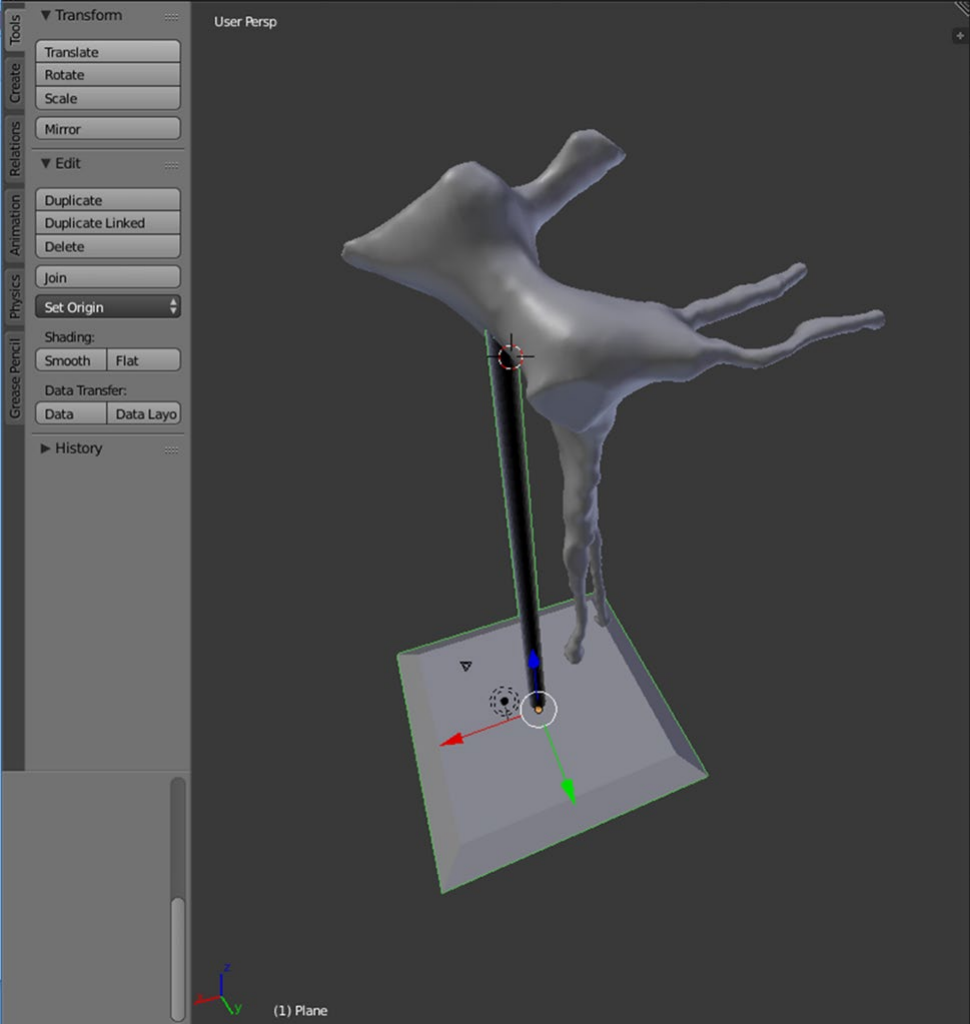
Base and strut designed in the *Blender* program

**Fig. 4:**
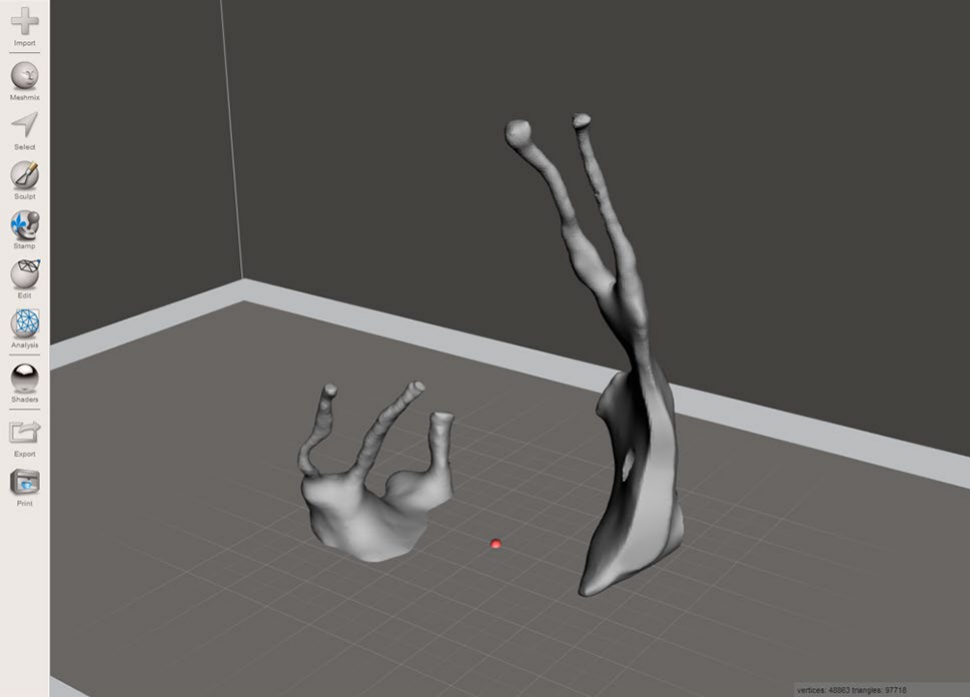
Bisected model, with each half orientated for printing in *Meshmixer*. This conformation minimizes the overhang angle of the greater and lesser palatine canals, the foramen rotundum, and the pterygoid and palato-vaginal canals. As a result, no support material is required to prop up these structures during printing. This reduces print time and avoids wastage of material

An open source software platform called *Meshmixer* (http://www.meshmixer.com Autodesk, Inc.) was used to edit and optimise the 3D render. It was used to smooth rough edges that arose during the pixel highlighting process. This ensured adequate definition of the foramina and fissures (Fig. 2). The scale of the model was increased to 2:1. *Blender* (http://www.blender.org, Blender Foundation) is an open source 3D modelling and rendering suite. The render was loaded into this program to design a stand for the printed model. A square frustum base with a cylindrical strut was created. The PPF render was loaded to create a recess that would accept the strut. This was performed by manipulation of individual vertices on the PPF render (Fig. 3). Due to the difficulty associated with printing overhanging features, the model was bisected in an oblique plane using *Meshmixer*. This resulted in two constituent parts, each with a wide, flat base, and a reduction in the overhang angle of the canals (Fig. 4).

To export the render to a 3D printer, it must first be converted into a G-code file. This contains instructions regarding multiple parameters, for example the movements that the printer nozzle must make to build up each layer of material. We used an open source program called *Slic3r Prusa Edition* (http://www.prusaprinters.org) for G-code conversion. Both parts of the model were imported into the program. Layer height—the crucial parameter that determines resolution—was set to 50 µm. First layer height was set to 100 µm to improve the bonding of the model to the print bed. Bonding was also facilitated by adjusting the print bed temperature to 55 °C. An extruder temperature of 215 °C was selected for the duration of the print. To avoid wastage of print filament, a cubic infill pattern with a density of 10% was incorporated into the G-code file. The vertical and horizontal shell perimeters—essentially the walls of the model—were set to 3 and 4 layers, respectively. No supports for the overhanging structures were added. Nozzle speed was calibrated to 20 mm/s (8 mm/s for the first layer).

**Fig. 5:**
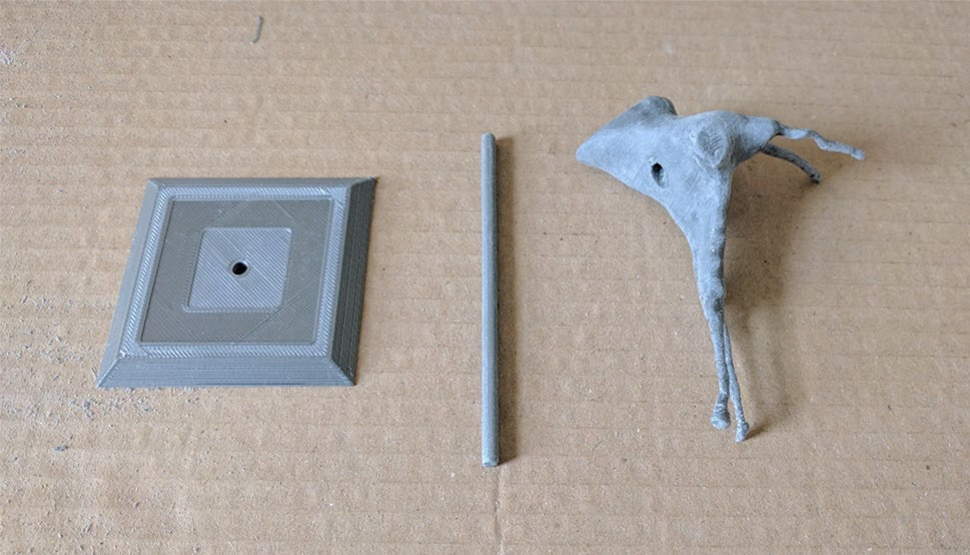
Base, strut, and model from *left* to *right*, respectively. The PPF has been filed and sanded to remove printing artefacts. The recess in the PPF that accepts the strut is clearly visible

**Fig. 6:**
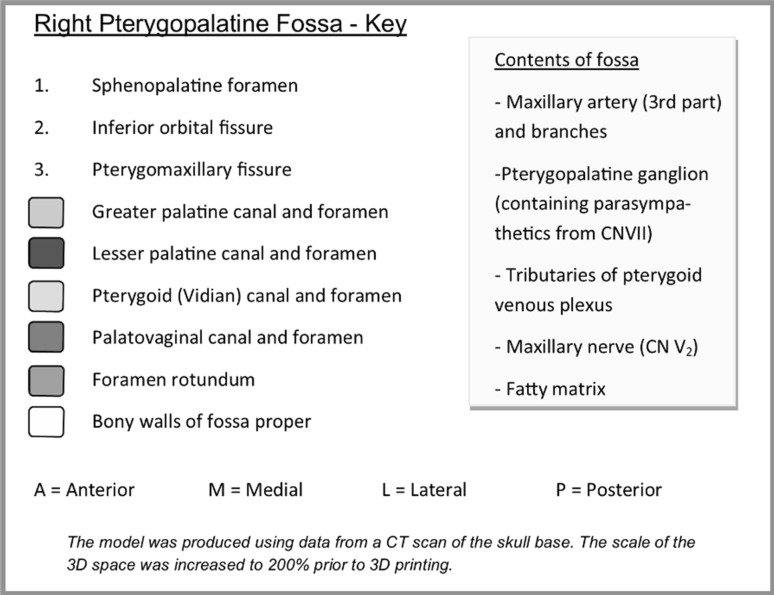
Key designed to accompany the model

**Fig. 7:**
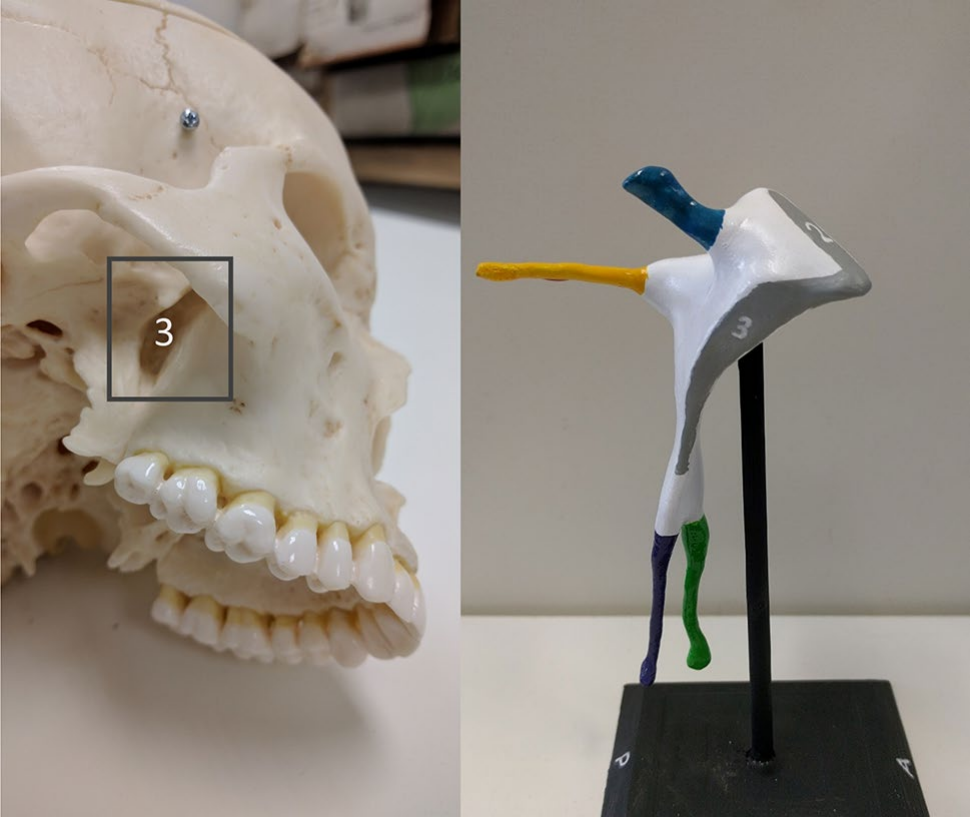
Illustration designed to accompany the model—the plastic skull is viewed form a right inferolateral aspect to reveal the pterygomaxillary fissure (*3*)

**Fig. 8:**
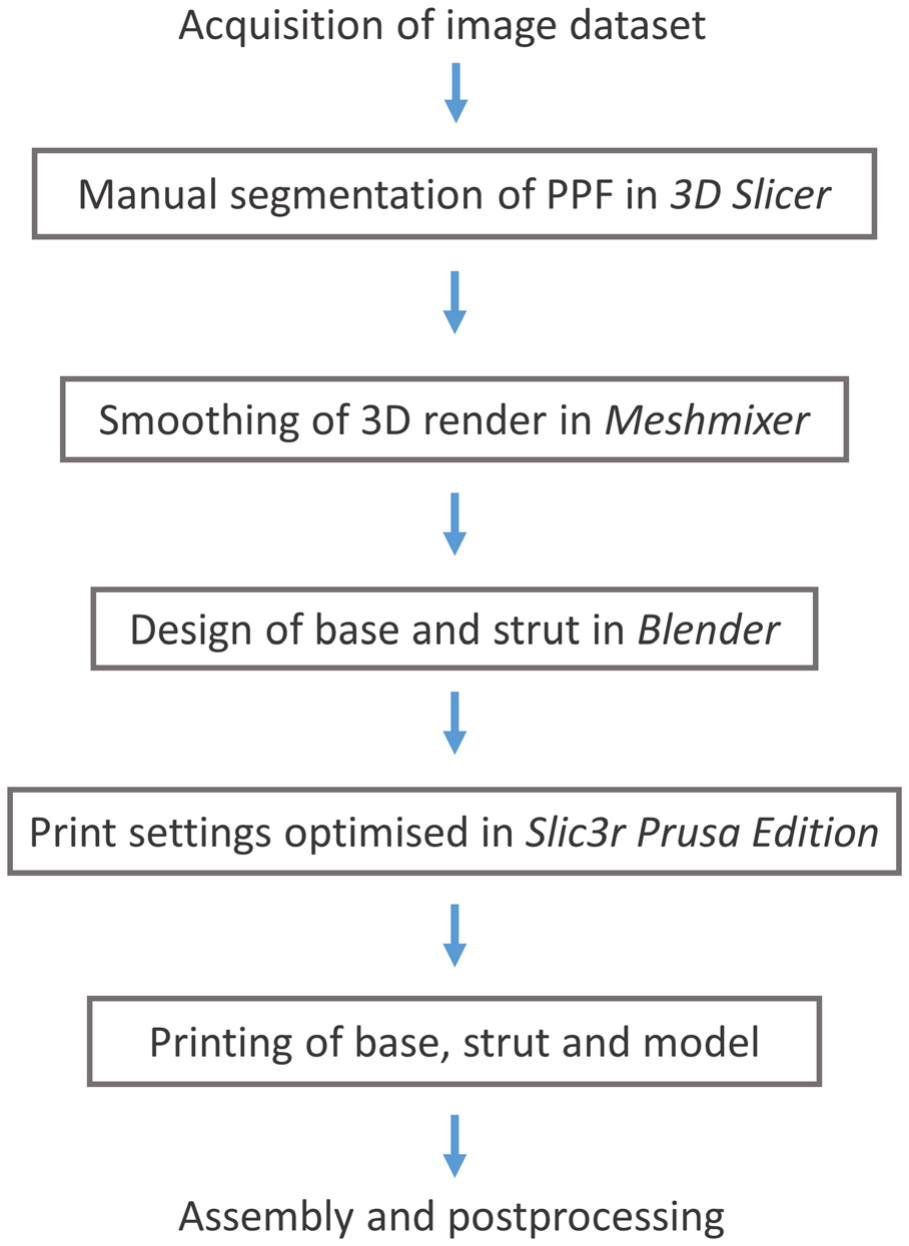
Flow diagram highlighting the design process

The model was then produced using an FDM 3D printer, a Prusa i3 MK2 (Prusa Research, http://www.prusa3d.com). The two parts of the PPF model were printed simultaneously, with the base and strut of the stand printed separately. Polylactic acid (PLA)—a biodegradable and eco-friendly thermoplastic polyester [9]—was used as the print material. The PLA filament has a diameter of 1.75 mm and costs £16.03 for a 1 kg reel. *Slic3r Prusa Edition* calculated the weight of material used for the model, strut, and base to be 8.12 g, based on the filament having a density of 1.24 g/cc, amounting to a cost of £0.13. Print time for the PPF proper was 6 h and 42 min, while the base and strut each took 34 min. Cyanoacrylate glue was used to join the two halves of the model, which was later filed and sanded to remove any roughened edges (Fig. 5). Acrylic paint was used to colour code the canals. A dark grey paint was used to represent the pterygomaxillary fissure, inferior orbital fissure, and sphenopalatine foramen. The completed model was mounted on the bespoke stand (Fig. 6). Finally, a key was designed to complement the colour coding. The model was designed to be used alongside a bony skull, to appreciate the position of the PPF in the skull base. A photograph of the lateral aspect of the model adjacent to the lateral aspect of the bony skull was added to the key to facilitate this (Fig. 7). The project workflow is summarised in Fig. 8.

## Results

**Fig. 9:**
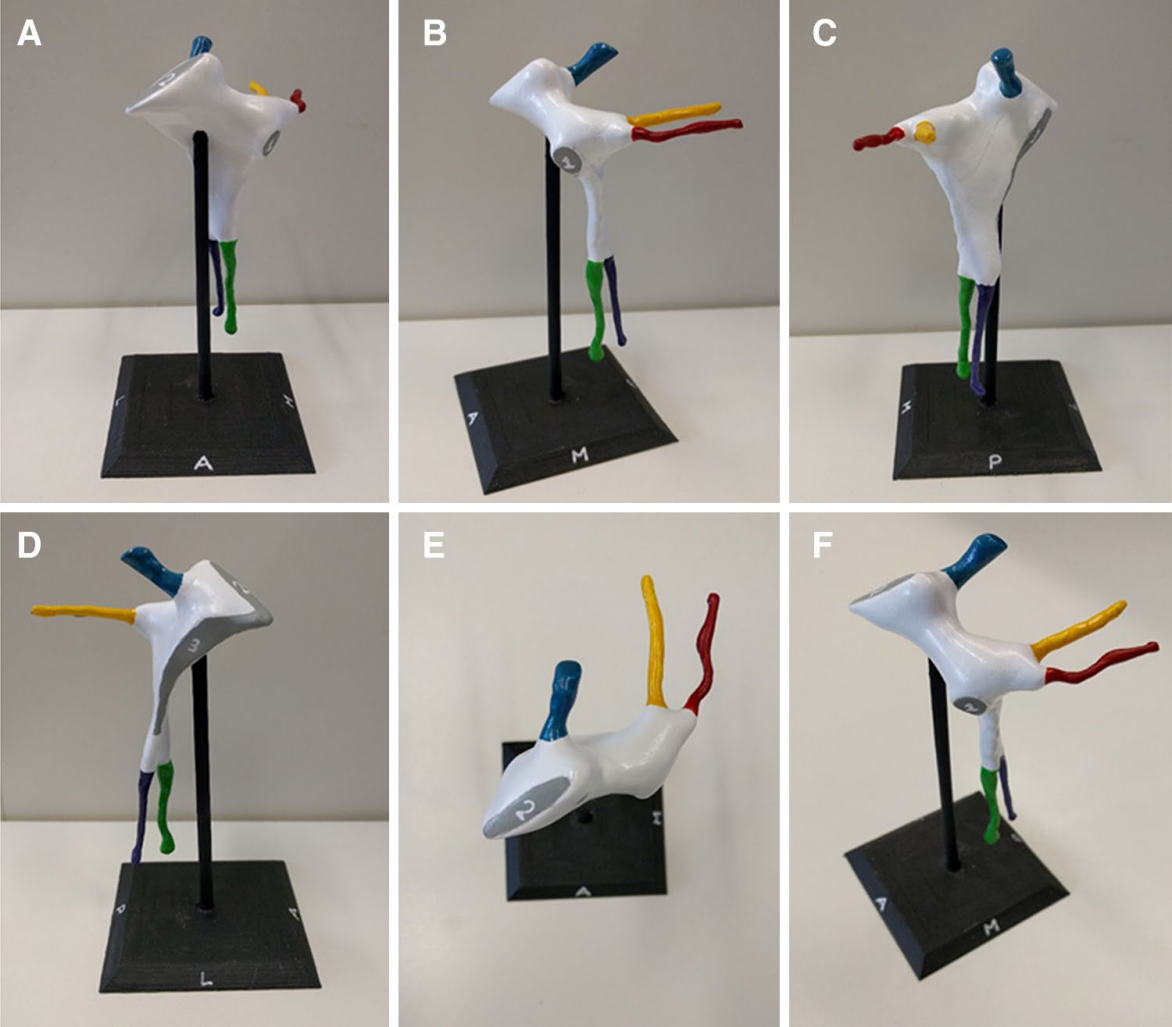
Completed model of the right PPF, mounted on the bespoke stand. **a** Anterior view. **b** Medial view. **c** Posterior view. **d** Lateral view. **e** Superior view. **f** Anterosuperomedial view. *Blue* foramen rotundum, *yellow* pterygoid canal, *red* palato-vaginal canal, *green* greater palatine canal, *pink* lesser palatine canal. *1* sphenopalatine foramen, *2* inferior orbital fissure, *3* pterygomaxillary fissure (colour figure online)

The completed model illustrates the shape of the fossa (coloured white) with associated canals: pterygoid canal (yellow), palato-vaginal canal (red), greater palatine canal (green), and lesser palatine canal (pink). The foramen rotundum (blue) is demonstrated by a canal-shaped projection, representing the passage from middle cranial fossa through the skull base. Finally, the sites of the sphenopalatine foramen, inferior orbital fissure, and pterygomaxillary fissure are coloured in grey and numbered 1–3, respectively. The model is mounted on the stand (black) in its anatomical position (Fig. 9). Orientation of the model is aided by the letters A (anterior), M (medial), L (lateral), and P (posterior), which are inscribed on each face of the stand. Interestingly, the model demonstrates the true shape of the PPF, which is more complex than a simple ‘inverse pyramid’. The 2:1 scale enables the user to appreciate the detail of the space and canals, whilst improving the structural integrity of the model. Prototype prints produced at a 1:1 scale resulted in a narrow and unstable lesser palatine canal that was prone to fracturing. The completed model, including the base, measures 11.4 cm in height and the base itself is 5.9 × 5.9 cm. Although we experimented with larger models, we felt that increasing the size further prohibited the user from mentally visualizing the space ‘in situ’.

## Discussion

Anatomical textbooks often attempt to explain the PPF with the aid of 2D schematics—this does not allow one to truly appreciate the 3D shape of the space. Our model, when used in conjunction with a bony skull, enables the user to visualise it directly. The benefits of physical 3D models have been highlighted by Preece et al., who analysed their effect on student learning. The ability of students to identify anatomical structures on an MRI of an equine foot was significantly greater if they had used a 3D model, compared to textbooks or computer models. Model use was also associated with a greater degree of student confidence [15]. Evidence of the effectiveness of 3D printed models in the classroom is now beginning to emerge. Lim et al. [10] conducted a randomized controlled trial comparing the use of 3D printed cardiac models with cadaveric prosections in the teaching of undergraduate medical students. Students using only the 3D printed models achieved significantly higher post-test scores than those using either both prosections and models, or prosections alone. Further studies to validate the use of 3D printed models in medical education are essential if they are to be integrated into curricula. Undergraduate medical students at our institution, who learn anatomy via full body cadaveric dissection, are required to know the structures passing through the PPF, and therefore, this model would be an invaluable teaching adjunct. However, we believe that this model would also be of particular interest to postgraduates in ENT, neurosurgery, ophthalmology and radiology, all of whom must have a thorough understanding of the space and its relationships.

Although not a new technology, advancements in this field have resulted in the mass production of affordable ‘desktop’ 3D printers. Small departments and even individuals can purchase their own printer—the Prusa i3 MK2 kit (Prusa Research, http://www.prusa3d.com **)** retails at £625. At this price, the purchaser must assemble the machine; however, it can be acquired pre-assembled at an increased cost. Crucially, this particular printer can work at a layer resolution of 50 µm, increasing the level of detail of the end product. Many similarly priced alternatives are available on the market. After the initial outlay for a printer, models can be replicated with minimal cost—the volume of PLA used in printing a single PPF model was only £0.13 worth of material. The use of open source software mitigates the need for expensive software packages. At present, desktop printers are unable to produce a multi-coloured, high-fidelity replica of an anatomical prosection, but they can produce accurate prints of simple structures such as isolated bones or conceptual models such as ours. The Z650 printer utilised by McMenamin et al., which retails at $65,000 [11], provides the means to reproduce an entire bank of anatomical prosections, albeit at a significantly increased cost. This technology is evolving rapidly: self-sufficient 3D printing anatomy laboratories may be a real possibility in the near future as the machinery and software required reduces in price. An added benefit is the ability to share G-code files. Rare anatomical variants detected on cross-sectional imaging could be rendered and the resulting digital model made available online for access worldwide. Consequently, this may be a viable alternative for the provision of anatomical teaching resources for medical schools and hospitals in low-income countries.

At present, we feel that desktop printers have much to add to the anatomy laboratory. Using a similar process as described here, anatomy departments could produce several models for use in teaching at a significantly reduced cost relative to those produced by commercial manufacturers, who often charge several hundred pounds for just one model. The versatility of 3D printing enables anatomical structures to be presented in a new light, giving alternative perspectives that can supplement traditional teaching using dissection or prosections.

## Limitations

Due to the low resolution of the CT scan, some detail was inevitably lost when highlighting pixels during manual segmentation. Smoothing of the render will also have resulted in a reduction in fidelity. Future studies could reduce the need for smoothing, whilst improving accuracy, using high-resolution CT or perhaps 7-Tesla MRI. Pragmatically, however, we feel that this would add little to the model’s value as a teaching tool. As the end product is a ‘negative space’ representation and hence a solid object, it does not directly demonstrate the course of the neurovascular structures passing through the PPF. The user will, however, be able to use the colour coded canals as a proxy for the passage of their respective contents. The boundaries of the fossa are not displayed in their entirety; for example, one cannot see the perpendicular plate of the palatine bone forming the medial border. We hope that the user will gain an appreciation of these relationships using the model in conjunction with a bony skull.

Another limitation is the steep learning curve associated with 3D printing. Several prototype prints failed due to either poor bonding to the heat bed, or problems associated with overhanging structures. Software packages such as *Slic3r* enable the incorporation of ‘supports’ into the G-code file, which prevent collapse of such features. The supports are removed following printing. We eliminated the need for supports by printing the model in two halves and adjusting nozzle speed for the more delicate parts. Anyone attempting to replicate our technique will undoubtedly encounter similar hurdles that can make the process labour-intensive. Nevertheless, once the optimal parameters have been determined, minimal monitoring is required for subsequent prints. The operator needs only to load the filament and initiate the print job. We believe that the process of prototyping models will become more efficient as the user gains experience and the technology more streamlined. Having a trained 3D printing technician may be an important addition for the anatomy laboratory of the future.

## Conclusion

We have described the creation of a cost-effective 3D model of the pterygopalatine fossa. By utilising a negative space representation of the fossa, we have presented the structure in a new light. We hope that others, through replication of our technique, will be able to develop innovative models of complex anatomical structures. Ongoing work aims to test the educational value of this particular model in the classroom.
